# Supplementary material for: Modeling glioblastoma heterogeneity as a dynamic network of cell states
Source: Mol Syst Biol. 2021 Sep 16;17(9):e10105. doi: 10.15252/msb.202010105 (PMC8444284; doi:10.15252/msb.202010105)
Supplement: Supplementary file 6 — Source Data for Figure 5 [file MSB-17-e10105-s004.zip › Figure5A_sourcedata/GSEA_3017/nosigdb_stateF.GseaPreranked.1622795015644/LARSSON_STATE_4.html]

Details for gene set LARSSON\_STATE\_4[GSEA]

|  || Dataset | state33017 |
| Phenotype | NoPhenotypeAvailable |
| Upregulated in class | na\_neg |
| GeneSet | LARSSON\_STATE\_4 |
| Enrichment Score (ES) | -0.889409 |
| Normalized Enrichment Score (NES) | -5.692013 |
| Nominal p-value | 0.0 |
| FDR q-value | 0.0 |
| FWER p-Value | 0.0 |
Table: GSEA Results Summary

  

Fig 1: Enrichment plot: LARSSON\_STATE\_4      
 Profile of the Running ES Score & Positions of GeneSet Members on the Rank Ordered List

  

| PROBE | GENE SYMBOL | GENE\_TITLE | RANK IN GENE LIST | RANK METRIC SCORE | RUNNING ES | CORE ENRICHMENT || 1 | E2F1 |  |  | 1580 | -0.281 | -0.7291 | No |
| 2 | MCM3 |  |  | 1738 | -0.335 | -0.7983 | No |
| 3 | RAN |  |  | 1772 | -0.350 | -0.8098 | No |
| 4 | CDC45 |  |  | 1836 | -0.382 | -0.8349 | No |
| 5 | ASF1B |  |  | 1897 | -0.419 | -0.8582 | No |
| 6 | UHRF1 |  |  | 1921 | -0.438 | -0.8641 | No |
| 7 | GINS2 |  |  | 1932 | -0.445 | -0.8640 | No |
| 8 | RFC2 |  |  | 1954 | -0.457 | -0.8688 | No |
| 9 | MCM4 |  |  | 1993 | -0.487 | -0.8812 | No |
| 10 | TK1 |  |  | 2003 | -0.497 | -0.8800 | No |
| 11 | SIVA1 |  |  | 2014 | -0.505 | -0.8792 | No |
| 12 | RRM1 |  |  | 2023 | -0.516 | -0.8774 | No |
| 13 | RANBP1 |  |  | 2024 | -0.517 | -0.8718 | No |
| 14 | STMN1 |  |  | 2063 | -0.567 | -0.8833 | Yes |
| 15 | PTMA |  |  | 2065 | -0.570 | -0.8776 | Yes |
| 16 | ZWINT |  |  | 2069 | -0.577 | -0.8728 | Yes |
| 17 | MND1 |  |  | 2074 | -0.586 | -0.8684 | Yes |
| 18 | LSM4 |  |  | 2076 | -0.587 | -0.8625 | Yes |
| 19 | CENPK |  |  | 2082 | -0.607 | -0.8583 | Yes |
| 20 | PHF19 |  |  | 2101 | -0.650 | -0.8597 | Yes |
| 21 | SNRPD1 |  |  | 2105 | -0.653 | -0.8540 | Yes |
| 22 | ALYREF |  |  | 2111 | -0.677 | -0.8490 | Yes |
| 23 | RPA3 |  |  | 2112 | -0.678 | -0.8418 | Yes |
| 24 | LSM3 |  |  | 2114 | -0.694 | -0.8348 | Yes |
| 25 | CKS2 |  |  | 2115 | -0.694 | -0.8273 | Yes |
| 26 | ANP32B |  |  | 2119 | -0.700 | -0.8212 | Yes |
| 27 | CENPM |  |  | 2125 | -0.709 | -0.8158 | Yes |
| 28 | HNRNPD |  |  | 2126 | -0.713 | -0.8082 | Yes |
| 29 | RAD51AP1 |  |  | 2127 | -0.715 | -0.8005 | Yes |
| 30 | BRCA1 |  |  | 2138 | -0.758 | -0.7970 | Yes |
| 31 | H2AFV |  |  | 2143 | -0.779 | -0.7904 | Yes |
| 32 | FBXO5 |  |  | 2144 | -0.779 | -0.7821 | Yes |
| 33 | GMNN |  |  | 2146 | -0.785 | -0.7741 | Yes |
| 34 | DEK |  |  | 2154 | -0.817 | -0.7685 | Yes |
| 35 | DUT |  |  | 2155 | -0.824 | -0.7597 | Yes |
| 36 | TUBB |  |  | 2157 | -0.831 | -0.7512 | Yes |
| 37 | NUCKS1 |  |  | 2158 | -0.832 | -0.7422 | Yes |
| 38 | LSM5 |  |  | 2159 | -0.835 | -0.7333 | Yes |
| 39 | MCM7 |  |  | 2160 | -0.839 | -0.7242 | Yes |
| 40 | DTYMK |  |  | 2168 | -0.858 | -0.7182 | Yes |
| 41 | MCM10 |  |  | 2169 | -0.858 | -0.7090 | Yes |
| 42 | PIMREG |  |  | 2175 | -0.876 | -0.7019 | Yes |
| 43 | KPNA2 |  |  | 2178 | -0.894 | -0.6932 | Yes |
| 44 | CHAF1A |  |  | 2182 | -0.903 | -0.6849 | Yes |
| 45 | KIF15 |  |  | 2185 | -0.915 | -0.6760 | Yes |
| 46 | KIF4A |  |  | 2186 | -0.921 | -0.6661 | Yes |
| 47 | ORC6 |  |  | 2188 | -0.937 | -0.6565 | Yes |
| 48 | SGO1 |  |  | 2190 | -0.942 | -0.6468 | Yes |
| 49 | CENPH |  |  | 2191 | -0.943 | -0.6367 | Yes |
| 50 | TUBA1B |  |  | 2193 | -0.947 | -0.6269 | Yes |
| 51 | LMNB1 |  |  | 2194 | -0.950 | -0.6167 | Yes |
| 52 | UBE2T |  |  | 2196 | -0.965 | -0.6068 | Yes |
| 53 | FANCI |  |  | 2198 | -0.966 | -0.5969 | Yes |
| 54 | CDKN3 |  |  | 2199 | -0.979 | -0.5863 | Yes |
| 55 | CKS1B |  |  | 2201 | -0.987 | -0.5762 | Yes |
| 56 | HIST1H1E |  |  | 2202 | -0.992 | -0.5655 | Yes |
| 57 | SGO2 |  |  | 2203 | -1.003 | -0.5547 | Yes |
| 58 | ESCO2 |  |  | 2204 | -1.004 | -0.5439 | Yes |
| 59 | SMC2 |  |  | 2205 | -1.006 | -0.5331 | Yes |
| 60 | EZH2 |  |  | 2207 | -1.017 | -0.5226 | Yes |
| 61 | DHFR |  |  | 2212 | -1.040 | -0.5133 | Yes |
| 62 | NUDT1 |  |  | 2214 | -1.054 | -0.5024 | Yes |
| 63 | MYBL2 |  |  | 2215 | -1.058 | -0.4910 | Yes |
| 64 | CLSPN |  |  | 2216 | -1.060 | -0.4796 | Yes |
| 65 | CENPN |  |  | 2217 | -1.079 | -0.4680 | Yes |
| 66 | UBE2C |  |  | 2218 | -1.081 | -0.4564 | Yes |
| 67 | CENPU |  |  | 2219 | -1.088 | -0.4447 | Yes |
| 68 | H2AFZ |  |  | 2220 | -1.090 | -0.4329 | Yes |
| 69 | HMGB1 |  |  | 2221 | -1.116 | -0.4209 | Yes |
| 70 | NCAPG |  |  | 2222 | -1.122 | -0.4089 | Yes |
| 71 | H2AFX |  |  | 2223 | -1.146 | -0.3965 | Yes |
| 72 | CCNB1 |  |  | 2224 | -1.148 | -0.3842 | Yes |
| 73 | CDK1 |  |  | 2225 | -1.153 | -0.3718 | Yes |
| 74 | PTTG1 |  |  | 2226 | -1.198 | -0.3589 | Yes |
| 75 | MAD2L1 |  |  | 2228 | -1.204 | -0.3464 | Yes |
| 76 | UBE2S |  |  | 2229 | -1.205 | -0.3334 | Yes |
| 77 | GTSE1 |  |  | 2230 | -1.220 | -0.3203 | Yes |
| 78 | DBF4 |  |  | 2232 | -1.247 | -0.3073 | Yes |
| 79 | HMGN2 |  |  | 2233 | -1.267 | -0.2937 | Yes |
| 80 | RRM2 |  |  | 2234 | -1.268 | -0.2801 | Yes |
| 81 | SMC4 |  |  | 2236 | -1.281 | -0.2668 | Yes |
| 82 | PBK |  |  | 2237 | -1.325 | -0.2525 | Yes |
| 83 | ATAD2 |  |  | 2238 | -1.381 | -0.2376 | Yes |
| 84 | TMPO |  |  | 2239 | -1.382 | -0.2228 | Yes |
| 85 | HMGB2 |  |  | 2241 | -1.469 | -0.2074 | Yes |
| 86 | BIRC5 |  |  | 2242 | -1.469 | -0.1916 | Yes |
| 87 | HIST1H1C |  |  | 2243 | -1.485 | -0.1756 | Yes |
| 88 | PCLAF |  |  | 2245 | -1.516 | -0.1598 | Yes |
| 89 | TOP2A |  |  | 2246 | -1.562 | -0.1430 | Yes |
| 90 | NUSAP1 |  |  | 2247 | -1.600 | -0.1258 | Yes |
| 91 | TPX2 |  |  | 2248 | -1.609 | -0.1085 | Yes |
| 92 | CENPF |  |  | 2249 | -1.691 | -0.0903 | Yes |
| 93 | HIST1H1D |  |  | 2250 | -1.720 | -0.0718 | Yes |
| 94 | HIST1H1B |  |  | 2251 | -1.796 | -0.0525 | Yes |
| 95 | MKI67 |  |  | 2252 | -1.892 | -0.0321 | Yes |
| 96 | HIST1H4C |  |  | 2253 | -2.984 | -0.0000 | Yes |
Table: GSEA details [plain text format]

  

Fig 2: LARSSON\_STATE\_4: Random ES distribution      
 Gene set null distribution of ES for **LARSSON\_STATE\_4**
